# Methylation-Associated Partial Down-Regulation of Mesothelin Causes Resistance to Anti-Mesothelin Immunotoxins in a Pancreatic Cancer Cell Line

**DOI:** 10.1371/journal.pone.0122462

**Published:** 2015-03-24

**Authors:** Kevin Hollevoet, Emily Mason-Osann, Fabian Müller, Ira Pastan

**Affiliations:** 1 Laboratory of Molecular Biology, Center for Cancer Research, National Cancer Institute, National Institutes of Health, Bethesda, MD, United States of America; 2 Laboratory for Therapeutic and Diagnostic Antibodies, Department of Pharmaceutical and Pharmacological Sciences, KU Leuven, Leuven University, Leuven, Belgium

## Abstract

Anti-mesothelin *Pseudomonas* exotoxin A-based recombinant immunotoxins (RITs) present a potential treatment modality for pancreatic ductal adenocarcinoma (PDAC). To study mechanisms of resistance, the sensitive PDAC cell line KLM-1 was intermittently exposed to the anti-mesothelin SS1-LR-GGS RIT. Surviving cells were resistant to various anti-mesothelin RITs (IC_50_s >1 μg/ml), including the novel de-immunized RG7787. These resistant KLM-1-R cells were equally sensitive to the anti-CD71 HB21(Fv)-PE40 RIT as KLM-1, indicating resistance was specific to anti-mesothelin RITs. Mesothelin gene expression was partially down-regulated in KLM-1-R, resulting in 5-fold lower surface protein levels and decreased cellular uptake of RG7787 compared to KLM-1. Bisulfite sequencing analysis found that the mesothelin promoter region was significantly more methylated in KLM-1-R (59 ± 3.6%) compared to KLM-1 (41 ± 4.8%), indicating hypermethylation as a mechanism of mesothelin downregulation. The DNA methyltransferase inhibitor 5-azacytidine restored original mesothelin surface expression to more than half in KLM-1-R and increased sensitivity to RG7787 (IC_50_ = 722.4 ± 232.6 ng/ml), although cells remained significantly less sensitive compared to parental KLM-1 cells (IC_50_ = 4.41 ± 0.38 ng/ml). Mesothelin cDNA introduction in KLM-1-R led to 5-fold higher surface protein levels and significantly higher RG7887 uptake compared to KLM-1. As a result, the original sensitivity to RG7787 was fully restored (IC_50_ = 4.49 ± 1.11 ng/ml). A significantly higher RG7787 uptake was thus required to reach the original cytotoxicity in resistant cells, hinting that intracellular RIT trafficking is also a limiting factor. RNA deep sequencing analysis of KLM-1 and KLM-1-R cells supported our experimental findings; compared to KLM-1, resistant cells displayed differential expression of genes linked to intracellular transport and an expression pattern that matched a more general hypermethylation status. In conclusion, resistance to anti-mesothelin RITs in KLM-1 is linked to a methylation-associated down-regulation of mesothelin, while aberrations in RIT trafficking could also play a role.

## Introduction

Our laboratory develops recombinant immunotoxins (RITs) for cancer treatment. Current RITs in clinical trials are composed of an antigen-binding Fv fused to a 38-kDa portion of *Pseudomonas* exotoxin A (PE) [1]. After receptor-mediated endocytosis, RITs are proteolytically processed, and PE is proposed to traffic to the trans-Golgi network and move by a retrograde pathway to endoplasmic reticulum, where it undergoes translocation to the cytoplasm [2]. Upon arrival in the cytosol, PE targets Elongation Factor-2 (EF-2). Mature EF-2 is produced by posttranslational modification of histidine 715 by the Diphthamide Biosynthesis proteins (DPH) 1–5 and 7 [3, 4]. This modified histidine (‘diphthamide’) is ADP-ribosylated by PE, which inactivates EF-2 and halts protein synthesis, eventually leading to programmed cell death [2].

We previously isolated and characterized several leukemic cell lines resistant to *Moxetumomab pasudotox* [5–7], an anti-CD22 RIT currently in phase III clinical trial (ClinicalTrials.gov Identifier: NCT01829711). These resistant cell lines show various aberrations in DPH expression, which prevent EF-2 ADP-ribosylation and protect cells from protein synthesis inhibition [5–7]. SS1(dsFv)-PE38 (SS1P), another RIT in clinical trials, targets mesothelin, a 40-kDa cell surface glycophosphatidylinositol (GPI)-anchored protein [8] that is highly expressed in several malignancies, including mesothelioma and pancreatic ductal adenocarcinoma (PDAC) [9–11]. SS1P has limited clinical activity as a single agent, primarily because of dose-limiting PE immunogenicity in patients [12, 13]. In response, SS1P has been combined with immune-depleting chemotherapeutics, resulting in unprecedented responses in patients with refractory advanced mesothelioma [14], and low-immunogenic RITs have been engineered in which many B- or T-cell epitopes and protease-sensitive regions of PE38 are removed. The latter resulted in a truncated and de-immunized 24-kDa toxin moiety (PE24) that has less reactivity with human anti-sera, is resistant to lysosomal degradation, and displays a decreased non-specific toxicity in rodent models *in vivo* [15–18]. In collaboration with Roche Innovation Center Penzberg, Germany, this PE24 backbone has been integrated into a novel anti-mesothelin RIT, called RG7787, by linking it to a humanized anti-mesothelin Fab, thereby increasing size and circulatory half-life [19].

We recently showed that RG7787 has significant activity in a PDAC xenograft model, which was established by grafting KLM-1 cells into immune deficient mice. RG7787 was also cytotoxic against several other PDAC cell lines, although *in vitro* cell killing was not absolute [19]. We previously reported that an imbalance between pro- and anti-apoptotic proteins protects cancer cells, including PDAC, from PE-induced cell death [20–22]. To gain insight into other mechanisms of resistance, the aim of this study was to isolate and characterize cells from KLM-1 that were resistant to anti-mesothelin RITs.

## Material and Methods

### Recombinant immunotoxins and reagents

Clinical-grade anti-mesothelin SS1P and anti-CD25 LMB-2 [anti-Tac(Fv)-PE38] were manufactured and provided by Advanced BioScience Laboratories, Inc. (Kensington, MD). huSS1(Fab)-LR-GGS-LO10-PE24 (RG7787) was supplied by Roche Innovation Center Penzberg, Germany under a Cooperative Research and Development Agreement (#2791). Anti-mesothelin SS1(dsFv)-LR-GGS-PE24 (SS1-LR-GGS) and the anti-CD71 immunotoxin HB21(Fv)-PE40 were produced in our laboratory, according to a standard protocol [23]. Both SS1-LR-GGS and RG7787 are re-engineered low-immunogenic versions of SS1P that consist of a PE24 fragment. Specific modifications include removing the bulk of PE domain II, leaving a furin cleavage site, and adding a Gly–Gly–Ser (GGS)-based peptide linker after the furin cleavage site. RG7787 is further optimized for clinical use by replacing the mouse anti-mesothelin Fv (SS1) with a humanized Fab (huSS1), to increase size and therefore circulatory half-life, and by introducing seven mutations (R505A, R427A, R490A, R467A, D463A, R456A, and R538A) in the catalytic domain III of PE to silence B-cell epitopes [19]. 5-azacytidine (AZA) (Sigma) is a DNA methyltransferase inhibitor, and was dissolved in RPMI-1640 medium.

### Cell culture

PDAC cell line KLM-1 [24] was maintained in RPMI-1640 and provided in 2011 by Dr. Udo Rudloff (NCI, Bethesda, MD), who originally obtained the cell line from the RIKEN Cell Bank. Epidermoid cancer A431 cell line was maintained in DMEM and donated in 1982 by Dr. George Todaro (NCI, Bethesda, MD), who originally isolated the cell line [25]. Media were supplemented with 10% FBS, 2 mM L-glutamine, 1 mM sodium pyruvate, 100 U/ml penicillin and 100 μg/ml streptomycin (Invitrogen). To isolate resistant cells, 3 x 10^5^ KLM-1 cells were seeded in a 6-well plate and incubated with 1 μg/ml SS1-LR-GGS for 72 hrs, which killed over 90% of the cells (S1 Fig.). Residual cells were expanded for 5 weeks in RIT-free cell medium, after which a second round of selection was performed similarly with 1 μg/ml SS1-LR-GGS, resulting in KLM-1-R. These cells were expanded in RIT-free medium and stored viably frozen in N_2_ for further studies. Cell line identities were verified using short tandem repeat analysis (NCI, Frederick, MD). All cells were maintained at 37°C in a humidified incubator with 5% CO_2_.

### Cell proliferation, cell death and protein synthesis inhibition assays

KLM-1 and KLM-1-R cell growth was compared by counting viable cells with a Cellometer Vision (Nexcelom). Dead cells were excluded using Trypan blue staining, and each time point was counted in triplicate. To evaluate treatment effect, RITs were added approximately 16 hrs after seeding of the cells in a 6- or 96-well plate. Growth inhibition was evaluated by measuring ATP levels with the Cell Titer-Glo Luminescent Cell Viability assay (Promega). Values were normalized between controls of 1 μM staurosporine (Sigma-Aldrich) and buffer (Dulbecco’s phosphate buffered saline without Ca and Mg (D-PBS), Quality Biological, Inc.) containing 0.2% human serum albumin (Division of Veterinary Resources, NIH, Bethesda, MD) or medium. Bright-field pictures were taken on a Zeiss microscope with a 10X EC Plan-NeoFluar objective using the AxioCam MRc camera and the AxioVision 4.7.2 acquisition software. Cell death was evaluated using the Annexin V-PE Apoptosis Detection Kit I (BD Pharmingen), according to manufacturer’s instructions. Apoptotic cells were considered Annexin V-positive, as determined by gating the untreated cells. Protein synthesis inhibition was quantified by measuring [^3^H]leucine (Perkin Elmer) incorporation as done previously [22]. Values are presented relative to controls of D-PBS 0.2% human serum albumin and 100 μg/ml cycloheximide- (Sigma-Aldrich) treated controls.

### Real-time RT-qPCR

RNA was isolated and purified from cells using the RNEasy kit (Qiagen), reverse transcription was done with the QuantiTect Reverse Transcription kit (Qiagen), and amplification with the QuantiFast SYBR Green PCR kit (Qiagen). Primer sequences for DPH1–5, DPH7, mesothelin, and β-actin are shown in S1 Table. Real-time RT-qPCR was performed on an ABI HT 7900 RT-PCR machine, analyzed using the comparative C_T_ method (ΔΔ*C*_T_) method with SDS manager (Applied Biosystems) and normalized for the endogenous β-actin.

### Surface protein expression by flow cytometry

Mouse anti-human mesothelin (MN; Rockland Immunochemicals, Inc.) and R-PE anti-human CD71 (Biolegend) were used to evaluate surface protein expression. A mouse IgG-R-PE isotype control (BD Biosciences) was used as a negative control for mesothelin staining. Anti-mesothelin and the isotype control were stained with a secondary goat anti-mouse IgG-R-PE (1:250 dilution, Jackson ImmunoResearch Laboratories, Inc.). Fluorescence intensity was analyzed by flow cytometry on a FACSCalibur. QuantiBRITE R-PE beads (BD Pharmingen) were used to quantify the number of mesothelin-binding sites per cell.

### Shed mesothelin levels in cell medium

Soluble mesothelin levels in cell line medium were measured in duplicate with a mesothelin Meso Scale Assay (Morphotek, Inc.), using the electrochemiluminescence technology of Meso Scale Discovery. Cells were seeded in regular cell culture medium in a 12-well plate. After 20 hrs, media was collected, the volume was measured and number of cells per well counted. After centrifuging the medium, supernatants was stored at −80°C until analysis. Cell supernatant was diluted 50-fold and added to wells of a 96-well plate previously coated with capture antibody. The procedure was performed as previously described [26], and signals were measured on an MSD Discovery Workbench (Meso Scale Discovery). The amount of mesothelin shed by KLM-1 and KLM-1-R was calculated by multiplying the obtained mesothelin concentration with well volume, and standardized for number of counted cells per well.

### RG7787 cellular uptake

RITs were labeled with the Alexa Fluor 647 Labeling Kit (Invitrogen) for 3.5 hrs and purified according to manufacturer’s instructions. Harvested cells were incubated for 30, 75 and 150 min at 37°C with a saturating 2 μg/ml of SS1P-Alexa647 or RG7787-Alexa647 and processed as previously described [22]. Fluorescence intensity was analyzed on a FACSCalibur. Uptake was expressed in number of internalized RG7787 molecules, which was calculated by assuming the RG7787-Alexa647 geomean surface expression of KLM-1 equal to 60 x 10^3^ RG7787 molecules (= surface mesothelin binding sites per KLM-1 cell, as evaluated by flow cytometry and QuantiBRITE R-PE beads).

### Methylation analysis

Evaluation of the methylation status of a region in the mesothelin gene promoter site was done by EpigenDx (Hopkinton, MA). Bisulfite modification was executed using the Zymo Research EZ Methylation Gold kit. Genomic DNA (500 ng) was used for bisulfite modification, followed by PCR amplification using Hot-Star Taq Polymerase (Qiagen). Pyrosequencing was performed using the PSQ HS 96 Pyrosequencing System (Qiagen). The analyzed region was chosen based on available primers (ADS2475-RS1 and ADS2475-RS2) at EpigenDx, which covered a 147-bp region upstream of the mesothelin transcription start site (chr16:808890-808742; ENST00000563941). Methylation quantification was performed with PyroQCpG software, which calculates for each single CpG the ratio between its methylated and non-methylated form, resulting in the average percentage of methylation degree.

### Mesothelin transfection in KLM-1-R

KLM-1-R cells were transfected by Lipofectamine (Invitrogen) with control pcDNA3.1(+) (Invitrogen) or pMH107, a pcDNA3.1(+) vector with full-length mesothelin cDNA and Geneticin as selectable marker [27]. Cells were transfected for three days, selected with 6 μg/ml Geneticin, and maintained in regular RPMI medium with 3 μg/ml Geneticin. Single cell sorting with a FACSVantage SE (BD Biosciences) and subsequent subsequent expansion resulted in the monoclonal cell line KLM-1-R-*Msln* that highly and homogeneously expressed mesothelin on its surface.

### Toxin-induced ADP-ribosylation of EF-2

Cells were lysed in RIPA buffer with protease inhibitors (Roche Applied Science), and 3 μg of cell lysate was incubated with ADP-ribosylation buffer [20 mM Tris-HCl (pH 7.4), 1 mM EDTA, and 50 mM DTT], 5 μM 6-Biotin-17-NAD (Trevigen) and 10 ng of RG7787 for various time points at 25°C. Samples were subjected to SDS/PAGE followed by Western blotting with streptavidin HRP conjugate (Invitrogen) to detect biotin ADP-ribosylated EF-2.

### Western blot

Cells were harvested, washed with D-PBS, and solubilized in RIPA buffer with protease inhibitors (Roche Applied Science). Protein concentrations were determined using a Coomassie Plus kit (Pierce). Equal amounts of protein were loaded onto NuPAGE 4%-12% Bis-Tris gels (Invitrogen) for SDS-PAGE and transferred to nitrocellulose membranes (Invitrogen). The following primary antibodies were used: mouse anti-MN (Rockland), rabbit anti-EF-2 #ab33208 (Abcam) and mouse anti-β-actin #8226 (Abcam). HRP-conjugated mouse secondary antibodies (Santa Cruz Biotechnology) were visualized with ECL or ECL Plus substrates (GE Healthcare). EF-2 protein levels were quantified and adjusted for β-actin with Image J [28].

### RNA deep sequencing and data analysis

Total RNA was extracted from KLM-1 and KLM-1-R cells using Qiagen RNEasy kit, and on-column DNA digestion (Qiagen) was performed according to manufacturer’s instruction. After RNA quality control with an Agilent Bioanalyzer, total RNA samples were sent for library construction and deep sequencing to the NCI core facility (Bethesda, MD). The raw sequences were quality controlled and aligned to RefSeq [29]. Raw counts were normalized and loaded into Qlucore Omics Explorer v_3.0(35) for differential expression analysis. Applying Qlucore’s variance filter, a list of the 989 most significantly changed genes was generated which we separated into up- and down-regulated genes. *Gene Set Enrichment Analysis* (GSEA) alignment was performed from within Qlucore. For alignment to Gene Ontology- (GO), Kyoto Encyclopedia of Genes and Genomes (KEGG)- and Reactome-pathways, the up- and down-regulated gene list was loaded into string-db.org [30] and p-values for datasets with significant changes were extracted.

### Statistical analysis

Experiments were typically performed independently at least twice, and representative or average data are displayed. Data are presented as mean ± standard error of measurement of replicate experiments. Applied statistics include Student t-tests or one-way ANOVA with Tukey’s multiple comparison tests. Statistical analysis and figure drafting was performed using GraphPad PRISM 6 (GraphPad Software, Inc.). A *p*-value of less than 0.05 was considered statistically significant, unless stated otherwise.

## Results

### KLM-1 cells isolated after SS1-LR-GGS incubation are resistant to anti-mesothelin RITs

**Fig 1:**
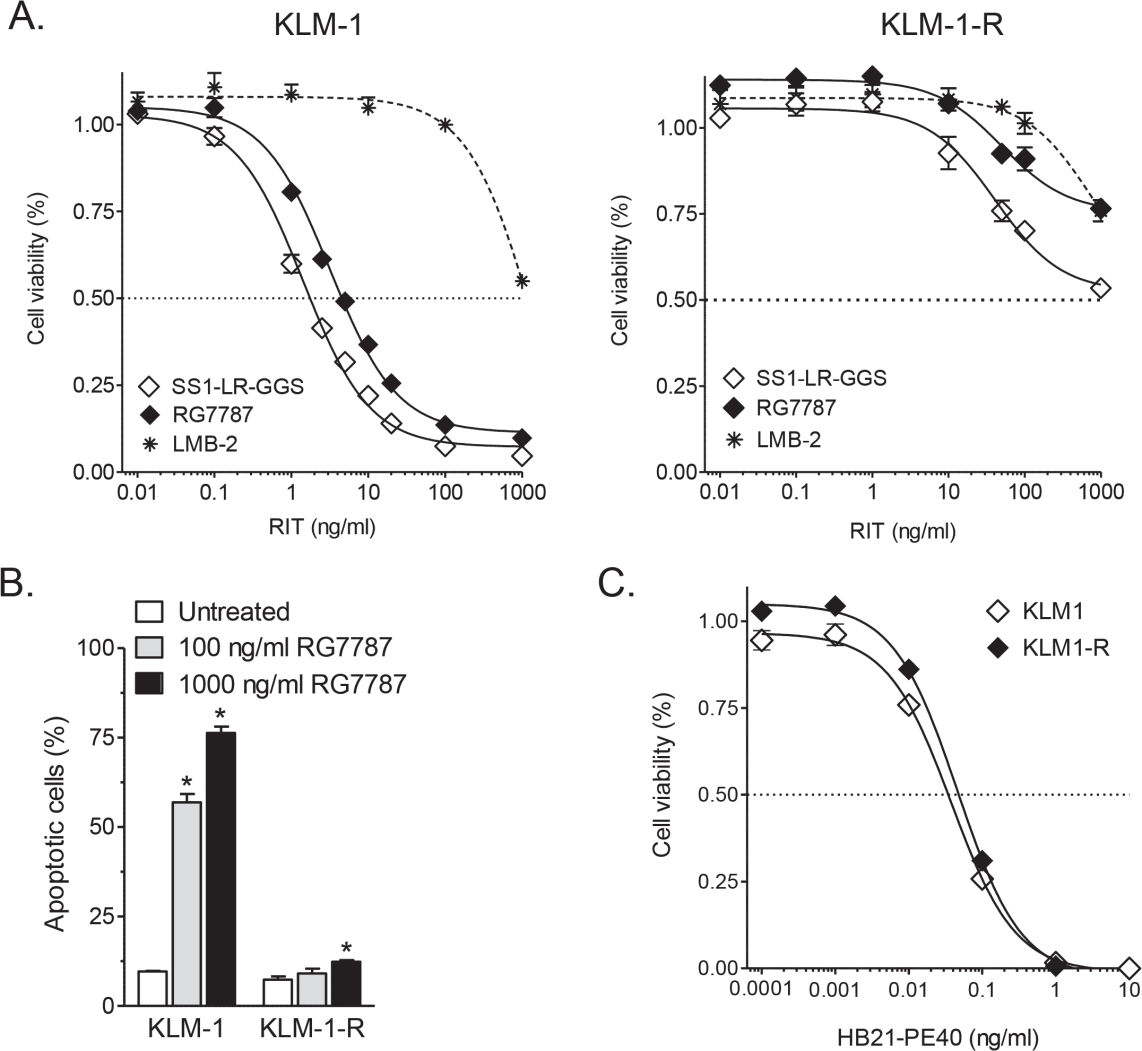
Activity of anti-mesothelin, anti-CD25 and anti-CD71 immunotoxins in KLM-1 and KLM-1-R. **A**: KLM-1 and resistant KLM-1 (KLM-1-R) cells were incubated for 72 hrs with the anti-mesothelin SS1-LR-GGS, RG7787 or anti-CD25 LMB-2 as a control. Growth inhibition was evaluated with an ATP cell viability assay. With IC_50_s below 10 ng/ml, KLM-1 is sensitive to the anti-mesothelin RITs, which is not the case for KLM-1-R (IC_50_s > 1 μg/ml). 1 μg/ml LMB-2 decreased cell viability, indicating that this RIT concentration induces non-specific uptake. **B**: KLM-1 and KLM-1-R cells were incubated for 72 hrs with 100 or 1000 ng/ml RG7787. Apoptosis was evaluated with the Annexin V-PE Apoptosis Detection Kit I. RG7787 induces a significant increase in apoptotic KLM-1 cells, whereas KLM-1-R cells shows no meaningful increase in apoptosis. **C**: KLM-1 and KLM-1-R cells were incubated for 72 hrs with HB21(Fv)-PE40. Growth inhibition was evaluated with an ATP cell viability assay. Both cell lines are highly sensitive to this RIT.

As described in the Methods section, KLM-1 cells were intermittently exposed with SS1-LR-GGS and surviving cells (KLM-1-R) were expanded in RIT-free medium. KLM-1 and KLM-1-R cells were indistinguishable by bright-field microscopy (S2A Fig.) and on flow cytometry forward and side scatter, indicating a similar cell size and granularity (S2B Fig.). Cell proliferation was compared by seeding 1 x 10^5^ cells on day 0, and counting viable cells daily for 6 days. KLM-1-R cells grew significantly faster than KLM-1 cells from day 2 on (*p* < 0.0001) (S2C Fig.). To evaluate the level of resistance, KLM-1 and KLM-1-R cells were incubated for 72 hrs with anti-mesothelin RITs. ATP viability assays showed that KLM-1 cells were sensitive to SS1-LR-GGS (IC_50_ = 1.73 ± 0.01 ng/ml) and RG7787 (IC_50_ = 4.41 ± 0.38 ng/ml), in agreement with previous findings [19]. In contrast, KLM-1-R cells were highly resistant to both RITs (IC_50_s > 1 μg/ml) (Fig. 1A) and SS1P (data not shown). In resistant cells, there was a small decrease in cell proliferation at RIT concentrations above a 100 ng/ml. As a control, we tested the activity of LMB-2, an anti-CD25 RIT [31] that does not bind KLM-1 cells, and observed a non-specific decrease at 1 μg/ml LMB-2. This indicates that the decrease at 1 μg/ml RG7787 in KLM-1-R could be attributed to non-specific uptake (Fig. 1A). The established resistance was stable; no change was observed when KLM-1-R cells were in RIT-free culture for several months (data not shown). Because ATP assays cannot differentiate between cell growth arrest and cell death [32], we evaluated response with the Annexin V-PE Apoptosis Detection kit. In contrast to KLM-1 (*p* < 0.0001), KLM-1-R cells showed no or limited apoptosis when treated for 72 hrs with 100 ng/ml (*p* = 0.33) or 1 μg/ml RG7787 (*p* = 0.02), as compared to untreated cells (Fig. 1B). These data confirm that KLM-1-R cells are highly resistant to the cell killing activity of anti-mesothelin RITs.

### Resistance in KLM-1-R cells is specific to anti-mesothelin RITs

To evaluate whether the resistance in KLM-1-R also applies to RITs targeted against other receptors on KLM-1-R cells, we tested HB21(Fv)-PE40 that targets CD71 [33]. CD71 is expressed at the surface at a similarly high level in KLM-1 and KLM-1-R (data not shown), and both cell lines had a similar sensitivity to HB21(Fv)-PE40 after a 72 hr incubation (Fig. 1C). To evaluate sensitivity to other therapeutics, cells were also treated for 72 hrs with paclitaxel, a mitotic inhibitor, and gemcitabine, a nucleoside analogue. Viability assays showed no difference in sensitivity to paclitaxel between KLM-1 and KLM-1-R (IC_50_ KLM-1 = 3.0 ± 1.6 ng/ml vs. IC_50_ KLM-1-R = 3.7 ± 1.7 ng/ml) (*p* = 0.80). KLM-1-R (IC_50_ = 44.2 ± 5.7 ng/ml) was 3-fold less sensitive to gemcitabine than KLM-1 (IC_50_ = 15.2 ± 1.9 ng/ml) (*p* = 0.04), which is much less pronounced than the resistance to RG7787. These results show that the resistance in KLM-1-R is not general and therefore specific to anti-mesothelin RITs.

### Mesothelin expression and RG7787 uptake is decreased in KLM-1-R

**Fig 2:**
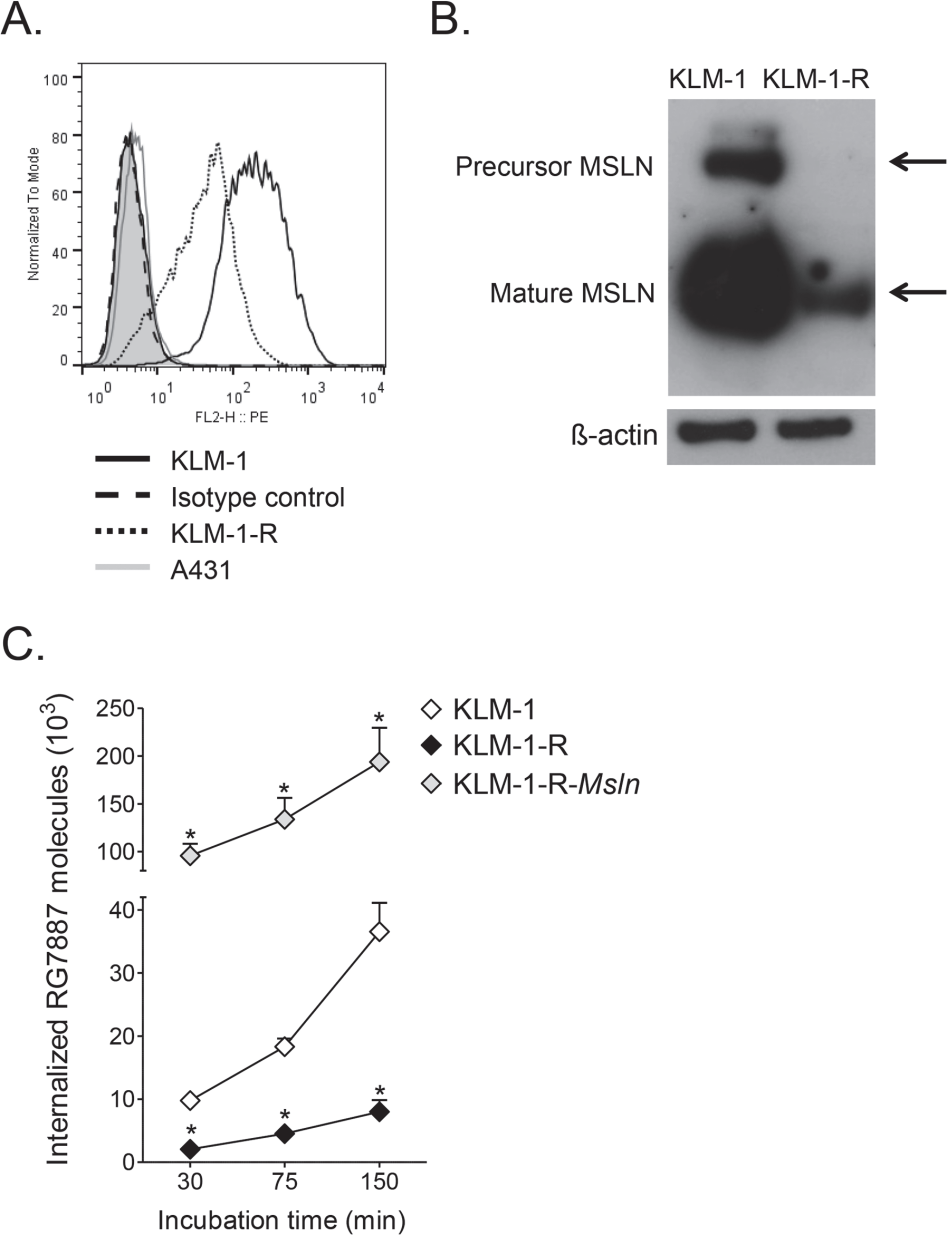
Mesothelin expression and RG7787 uptake is decreased in KLM-1-R. **A**: Surface mesothelin levels are 5-fold lower in resistant KLM-1 (KLM-1-R) compared to KLM-1. Mesothelin expression of KLM-1, KLM-1-R and mesothelin-negative A431 cells (negative control) were evaluated using flow cytometry. Filled histograms are secondary antibody controls. **B**: Whole mesothelin protein level is decreased in KLM-1-R. Precursor (72 kDa) and cleaved mature mesothelin (37 kDa) were present in KLM-1. In KLM-1-R, the cleaved portion was detected at low levels. Protein levels were probed in untreated KLM-1 and KLM-1-R cell lysate by Western blot. β-actin acts as loading control. **C**: At each time point, cellular uptake of RG7787-Alexa647 in KLM-1 is significantly higher than in KLM-1-R, and significantly lower than in KLM-1-R-*Msln* (transfected with mesothelin). Uptake was evaluated at 30, 75 and 150 min. Average geomean fluorescence intensities converted into amount of RG7787 molecules.

The first step in the RIT mechanism of action is binding to the targeted cell surface antigen followed by RIT internalization. Mesothelin surface expression, as evaluated by flow cytometry, was 4.9 ± 0.5-fold lower in KLM-1-R (12 x 10^3^ sites per cell) compared to KLM-1 (60 x 10^3^ sites per cell) (Fig. 2A). Flow cytometry displayed a homogeneous KLM-1-R cell population, suggesting a uniform decrease in mesothelin surface expression. Analysis of the mesothelin-negative A431 cells and use of an isotype antibody control confirmed that the mesothelin signal in KLM-1-R cells was specific. As expected, western blots showed that KLM-1 cells contained a major band of mature mesothelin at 37-kDa and a weaker precursor band at 72-kDa. The resistant cells had small amounts of mature mesothelin, but the precursor was not detected, likely due to its low abundance (Fig. 2B). Excess shedding of mesothelin in KLM-1-R could account for low mesothelin surface levels. Using the Meso Scale Assay, we found that shed mesothelin was 4.8 ± 0.03-fold lower in the media of KLM-1-R (40.7 ± 5.3 pg/10^5^ cells) compared to KLM-1 (149.0 ± 23.4 pg/10^5^ cells). In medium without cells (negative control), no mesothelin was detected. These data are consistent with the difference in surface levels and indicate that the low mesothelin on KLM-1-R is not due to increased shedding. To determine if decreased mesothelin was due to less mRNA, we performed RT-PCR and found that mesothelin RNA was 7.3 ± 4.3-fold lower in KLM-1-R (C_T_ = 24.1 ± 0.09) compared to KLM-1 (C_T_ = 21.54 ± 0.73). To evaluate whether the remaining mesothelin on the KLM-1-R surface could bind and internalize anti-mesothelin RITs, we evaluated the cellular internalization of RG7787-Alexa647. Uptake in KLM-1-R increased over time, but was significantly lower than KLM-1 at each time point (4- to 5-fold, *p* < 0.01). After 150 min incubation, e.g., KLM-1 internalized about 40 x 10^3^ RG7787 molecules, compared to only 8 x 10^3^ in KLM-1-R (Fig. 2C). These data demonstrate that KLM-1-R cells have a partial down-regulation in mesothelin and therefore internalize significantly less RG7787, providing a potential explanation for the observed resistance.

### AZA partially restores mesothelin surface expression in KLM-1-R with limited effect on sensitivity to RG7787

**Fig 3:**
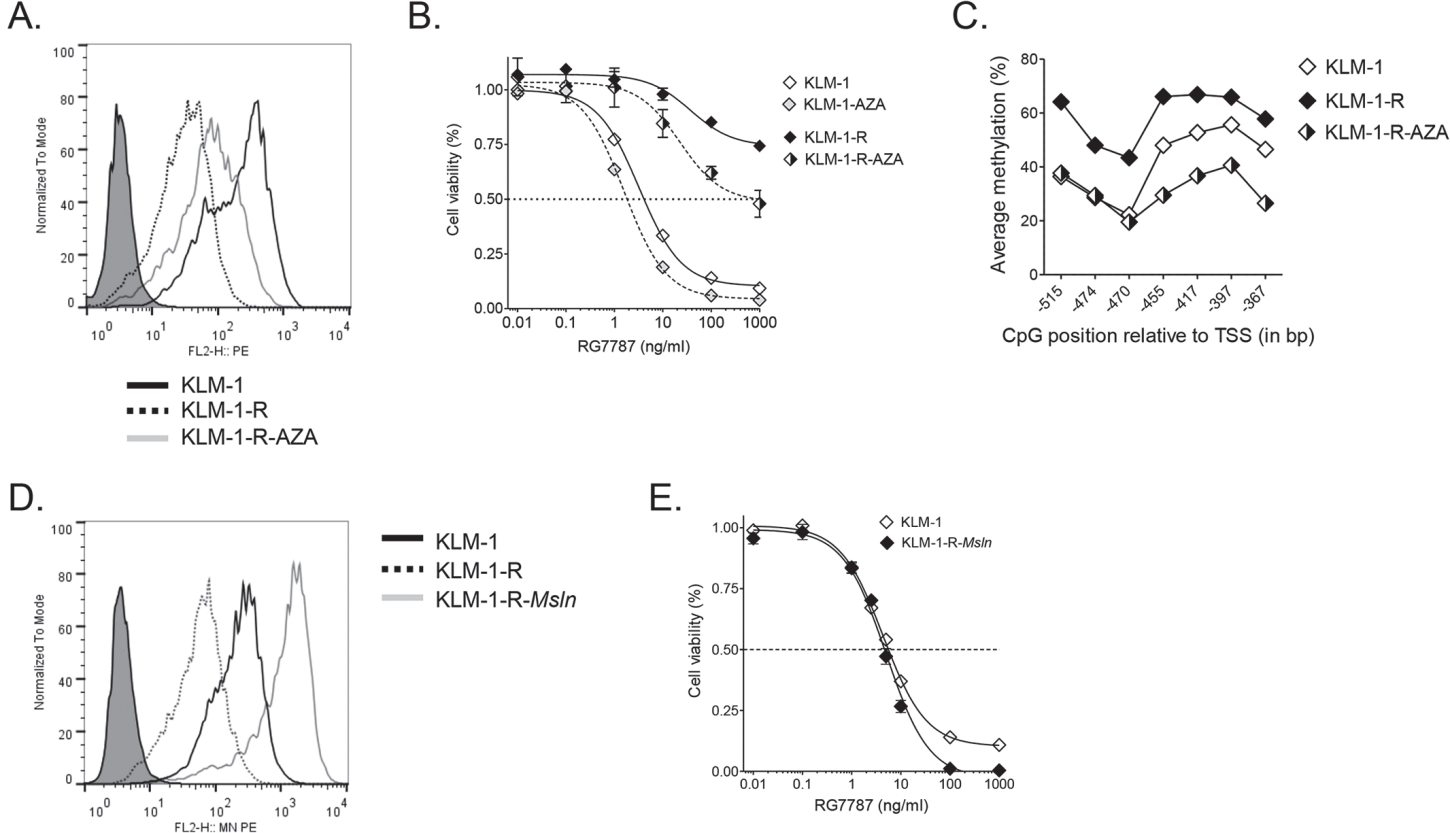
Mesothelin downregulation is associated with CpG hypermethylation and mesothelin transfection restores sensitivity to of KLM-1-R to RG7787. **A**: AZA leads to a 2.8-fold increase of mesothelin surface expression in resistant KLM-1 (KLM-1-R). Flow cytometry histogram of KLM-1, KLM-1-R, and KLM-1-R-AZA. Filled histograms represent secondary antibody controls. **B**: Three weeks of incubation with AZA, a DNA methyltransferase inhibitor, increases sensitivity to RG7787. KLM-1, KLM-1-R, and the AZA-treated cells (KLM-1-AZA and KLM-1-R-AZA) were treated for 72 hrs with RG7787. Growth inhibition was evaluated with an ATP cell viability assay. Dotted lines represent AZA-treated cells. **C**: CpGs in a region upstream of the mesothelin transcription start site are more methylated in KLM-1-R than in KLM-1. Three weeks of incubation with AZA decreases methylation in KLM-1-R cells. The analyzed region is located at chr16:808890-808742 and spans 147 bp and seven CpGs. **D**: Mesothelin transfection in KLM-1-R results in significant overexpression of mesothelin compared to KLM-1 (5-fold) and KLM-1-R (23-fold). Flow cytometry surface mesothelin levels in KLM-1, KLM-1-R and mesothelin-transfected resistant cells (KLM-1-R-*Msln*). **E**: Mesothelin overexpression in KLM-1-R restores sensitivity to RG7787. KLM-1 and KLM-1-R-*Msln* cells were incubated for 72 hrs with RG7787. Growth inhibition was evaluated with an ATP cell viability assay.

Mesothelin expression can be silenced by DNA methylation of CpG sites in its promoter region [34–37]. AZA, a DNA methyltransferase inhibitor, can reverse such hypermethylation. We gave cells 500 nM AZA daily for 3 weeks, and found that mesothelin expression was increased in KLM-1-R (KLM-1-R-AZA) by about 3-fold, up to 34 x 10^3^ sites per cell, which is still less than the 60 x 10^3^ sites per cell in KLM-1 (Fig. 3A). AZA improved the sensitivity to RG7787 in KLM-1 and KLM-1-R, although the latter remained highly resistant after a 72 hr incubation (IC_50_ = 722.4 ± 232.6 ng/ml) (Fig. 3B). These data link mesothelin down-regulation to hypermethylation.

### CpGs in mesothelin promoter region are hypermethylated in KLM-1-R

To confirm that the mesothelin gene is indeed subject to hypermethylation in KLM-1-R, we performed an exploratory analysis of the methylation status of seven CpGs in the mesothelin promoter region (chr16:808890-808742, S3 Fig.) using bisulfite sequencing. This 147-bp site region was selected based on the primers available at EpigenDx. Results demonstrated that these CpGs had a significantly higher methylation in KLM-1-R (59 ± 3.6%) compared to KLM-1 (41 ± 4.8%) (*p* < 0.05) (Fig. 3C). Treatment with AZA brought the methylation levels in KLM-1-R back to those of KLM-1 (p > 0.05). These data further support that mesothelin downregulation in KLM-1-R is associated with hypermethylation of the mesothelin gene.

### Overexpression of mesothelin in KLM-1-R restores sensitivity to RG7787

To restore mesothelin expression in KLM-1-R, we introduced full-length mesothelin cDNA (KLM-1-R-*Msln*). Surface mesothelin expression of KLM-1-R-*Msln* was 22.6 ± 5.3-fold higher than that of KLM-1-R, and exceeded the original expression in KLM-1 by 5.3 ± 1.3-fold, resulting in about 300 x 10^3^ sites per cell (Fig. 3D). As a consequence, the uptake of RG7787-Alexa647 in KLM-1-R-*Msln* at each time point was significantly higher than in KLM-1 (5- to 10-fold, *p* < 0.05) and KLM-1-R (24- to 46-fold, *p* < 0.001) (Fig. 2C). After a 72 hr incubation, RG7787 had a similar IC_50_ in KLM-1-R-*Msln* (4.49 ± 1.11 ng/ml) as in KLM-1 (4.41 ± 0.38 ng/ml) (*p* = 0.80). However, KLM-1-R-*Msln* was more sensitive than KLM-1 at higher RG7787 concentrations, with cell viability decreasing to zero at 100 ng/ml (Fig. 3E). Introduction of control pcDNA3.1(+) had no effect on mesothelin levels or resistance to RG7787 (data not shown). These data confirm that the resistance in KLM-1-R is linked to a decrease in mesothelin. In order to achieve a similar level of sensitivity to RG7787 seen in KLM-1, KLM-1-R requires significantly higher mesothelin levels than KLM-1. This finding hints that resistance could also be tied to inefficient trafficking of RG7787 to the cytosol, or that protein synthesis inhibition is affected in KLM-1-R.

### Protein synthesis inhibition by RG7787 is limited in KLM-1-R, EF-2 ADP ribosylation is intact

**Fig 4:**
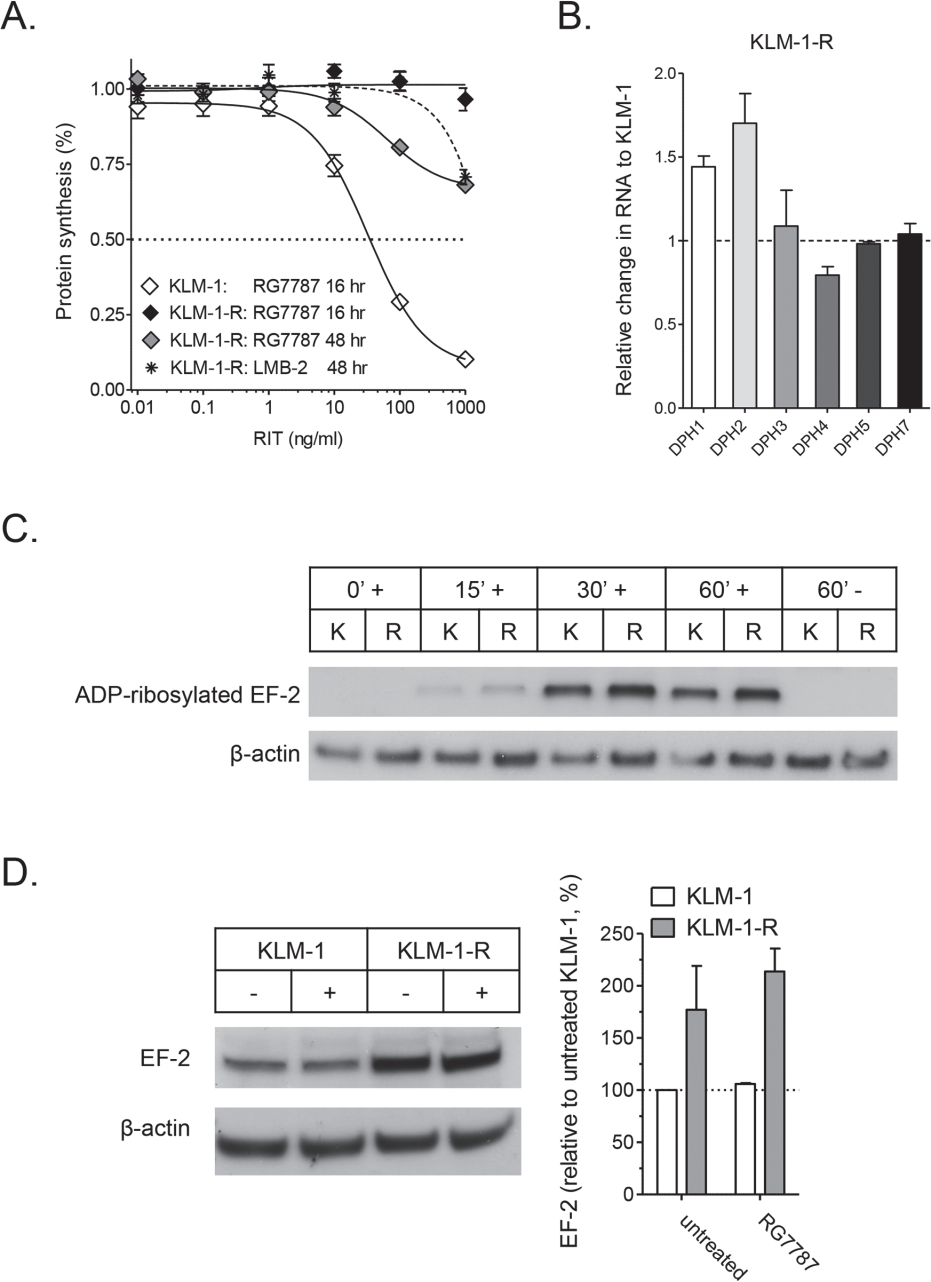
Protein synthesis inhibition and EF-2 ADP-ribosylation in KLM-1-R. **A**: Protein synthesis inhibition by RG7787 is limited in resistant KLM-1 (KLM-1-R). KLM-1 was incubated for 16 hrs with RG7787, and KLM-1-R for 16 and 48 hrs with RG7787 and anti-CD25 LMB-2. RG7787 induces a dose-dependent protein synthesis inhibition in KLM-1-R, which is absent in KLM-1-R. After 48 hrs, RGG778 induces some decrease in protein synthesis in KLM-1-R, which is also the case with LMB-2. Protein synthesis inhibition was evaluated by measuring [^3^H]leucine incorporation. **B**: Diphthamide Biosynthesis Protein (DPH) genes expression is not down-regulated in KLM-1-R, compared to KLM-1. Expression levels were evaluated with real time RT-PCR, standardized for ß-actin and presented relative to KLM-1 **C**: EF-2 ADP-ribosylation is functional in KLM-1-R. RIT-induced EF-2 ADP-ribosylation was evaluated by incubating cell lysate with ADP-ribosylation buffer, 6-Biotin-17-NAD and 10 ng of RG7787 for 0, 15, 30 and 60 min at 25°C. Samples were subjected to SDS/PAGE followed by Western blotting with streptavidin HRP conjugate to detect biotin ADP-ribosylated EF-2. The 0 min time point and the sample without RG7787 are negative controls. **D**: EF-2 protein levels are on average 2-fold higher in KLM-1-R compared to KLM-1. Western blot was done on cell lysate of KLM-1 and KLM-1-R. β-actin acts as loading control. Protein levels were quantified and adjusted for β-actin levels with Image J. K: KLM-1, R: KLM-1-R,—no RG7787, + with RG7787.

Protein synthesis inhibition is initiated after the toxin traffics from the cell surface to the cytosol and inactivates EF-2 by ADP-ribosylation. KLM-1 cells were incubated with RG7787 for 16 hrs, and KLM-1-R cells for 16 and 48 hrs, after which protein synthesis was examined by measuring [^3^H]leucine incorporation (Fig. 4A). After 16 hrs, RG7787 induced a dose-dependent decrease in protein synthesis in KLM-1, but not in KLM-1-R. After 48 hrs, KLM-1-R showed a small decrease in protein synthesis at the higher concentrations of RG7787, reminiscent of the growth inhibition observed with the ATP assay (Fig. 1A). LMB-2 caused protein synthesis inhibition above 100 ng/ml after 48 hrs, confirming that the protein synthesis inhibition in KLM-1-R at higher RG7787 concentrations is in part attributable to non-specific uptake. Next, we evaluated whether the dismal protein synthesis inhibition by RG7787 in KLM-1-R was due to a problem with EF-2 ADP ribosylation. The toxin inactivates EF-2 by ADP-ribosylation of the diphthamide residue on EF-2, which requires the activity of enzymes DPH1–5 and 7 [3]. We measured expression of these six diphthamide genes by RT-qPCR and found no meaningful decrease in KLM-1-R, compared to KLM-1 cells (Fig. 4B). To investigate the status of EF-2 in KLM-1-R, we examined EF-2 protein levels and the ability of RG7787 to ADP-ribosylate EF-2 in cell-free extracts at different times of RG7787 incubation. On average, EF-2 levels were 2-fold higher in KLM-1-R cells compared to KLM-1 cells (Fig. 4D). At each time point, the amount of EF-2 that was ADP-ribosylated by RG7787 was similar in KLM-1 and KLM-1-R (Fig. 4C). These data demonstrate that the dismal protein synthesis inhibition in KLM-1-R is not linked to downregulation of DPH enzymes or failure of the toxin to ADP-ribosylate EF-2. These data show that the anti-mesothelin RIT resistance is linked to events occurring upstream of protein synthesis inhibition, which is in agreement with the earlier findings that the resistant and KLM-1 cells were equally sensitive to the anti-CD71 HB21(Fv)-PE40.

### RNA deep sequencing gene expression analysis

We carried out RNA deep sequencing on KLM-1 and KLM-1-R cells and analyzed the data set using Qlucore. Applying the software’s algorithm for variance, we identified the top up- and down-regulated genes for KLM-1 versus KLM-1-R. The list of genes was separated in KLM-1-R’s up- (488 genes) and down- (501 genes) regulated genes, respectively (S2 Table). Similar to our earlier RT-PCR data, mesothelin (*MSLN*) was 9.08-fold down-regulated in KLM-1-R. To validate the dataset further, we checked for expression levels and fold-changes of common housekeeping genes and found them to be highly expressed (e.g. actin ∼0.5% of total counts) with no significant changes between sensitive and resistant cells (actin 1.117-fold, ribosomal protein L22 1.085-fold change). The RNA sequencing dataset was considered reliable as it confirmed the experimentally proven down-regulation of *MSLN* in KLM-1-R and displayed stability in housekeeping genes between the data sets.

### Functional analysis of RNA sequencing data reveals methylation-associated changes

The two sets of up- and down-regulated genes of KLM-1-R were separately applied to the GSEA database supplied by the Broad Institute [38]. GSEA sets with high similarity to our dataset were picked and applied to the whole unfiltered KLM-1/KLM-1-R data set of 25671 genes. One of the GSEA sets, “missiaglia_regulated_by_methylation_dn”, showed high similarity to our data. This GSEA set was originally generated by treating PDAC cell lines with AZA [39]. Of the 122 down-regulated genes in this GSEA, 97 (80%) were also down-regulated in the KLM-1-R cell population, whereas 20 genes (16%) were up-regulated and 5 genes (4%) were not overlapping (S4 Fig.). In accordance with above-described promoter methylation analysis of the *MSLN* gene, these data indicate a more general hypermethylated state in KLM-1-R.

### Protein network analysis highlights several clusters of differentially-expressed genes

The gene lists generated by Qlucore were next analyzed with the online tool STRING [30]. This tool clusters proteins based on databases from genomic contexts, high throughput experiments and co-expression, and implements a Pubmed text search. The resulting protein network can then be analyzed by GO annotations or by Reactome and KEGG pathway comparisons to screen for significances in differentially expressed gene sets. Up- and down-regulated genes were again kept separated in this analysis. Looking at the up-regulated genes in KLM-1-R, the largest sets showed genes that are involved in translation, transport and stress responses (Table 1). In accordance with an increased protein production, genes for unfolded protein response, tRNA-synthesis as well as metabolic RNA processes were up-regulated. An increase in these datasets resulted in highly significant *p*-values (< 0.001) for the GO-BL (gene ontology, biological function), KEGG, and Reactome pathway analyses. The dataset for down-regulated genes of KLM-1-R generally showed less clear patterns and lower significance levels, with several of them not reaching the *p* < 0.001 threshold for statistical significance (Table 1), including the transport-related datasets. Overall, the deep sequencing analyses of KLM-1 and KLM-1-R show profound changes in the expression profile of many cellular processes that are supportive of our earlier experimental observations.

**Table 1:** Significance level for differentially expressed gene sets from KLM-1 to KLM-1-R.

Up-regulated in KLM-1-R				Down-regulated in KLM-1-R			
Descriptive dataset name	Dataset reference	*p*-value*	No. of genes^§^	Descriptive dataset name	Dataset reference	*p*-value*	No. of genes^§^
Peptide chain elongation	Reactome	4,36E-91	63	Cell cycle process	GO-BL	5,89E-19	70
Translational elongation	GO-BL	1,78E-79	68	Response to stress	GO-BL	1,98E-17	133
Ribosome	KEGG	7,52E-62	50	Cell death	GO-BL	4,99E-14	74
Intracellular protein transport	GO-BL	2,09E-43	82	mRNA splicing—major pathway	Reactome	5,68E-11	20
Establishment of localization in cell	GO-BL	1,18E-37	117	mRNA splicing, via spliceosome	GO-BL	8,99E-10	24
Response to stress	GO-BL	1,64E-16	127	Cell cycle	KEGG	3,23E-09	19
Response to ER stress	GO-BL	9,68E-16	26	Endoplasmic reticulum-phagosome pathway	Reactome	5,89E-09	15
RNA metabolic process	GO-BL	3,90E-15	130	Proteasome	KEGG	2,54E-06	10
Translational elongation	GO-BL	4,95E-15	34	Activation of NF-κB in B cells	Reactome	3,11E-06	11
Aminoacyl-tRNA biosynthesis	KEGG	3,29E-11	12	Vesicle-mediated transport	GO-BL	1,75E-03	38
Focal adhesion	KEGG	3,49E-11	23	Lysosome	KEGG	2,11E-03	11
Positive regulation programmed cell death	GO-BL	9,84E-09	31	Expression of IFN-induced genes	Reactome	4,29E-03	7
Vesicle mediated tranport	GO-BL	1,68E-01	29	Lysosome	GO-CC	5,45E-03	21
				Actin cytoskeleton organization	GO-BL	5,96E-03	18
				Endosome	GO-CC	4,77E-01	19
Total input 478 genes (up-regulated)				Total input 489 genes (down-regulated)			

Table was generated by STRING using GO, KEGG, and Reactome databases, as indicated under dataset reference.

§Number of genes in our data set that are also present in the respective data-set list or pathway list.

*p-value represents the probability that 478 genes would show the distribution to match the same number of gene hits in the respective list. Datasets are ranked according to p-value. P < 0.001 is considered statistically significant.

## Discussion

To gain insight into resistance mechanisms to anti-mesothelin RITs, we isolated and characterized resistant cells from the PDAC cell line KLM-1. These KLM-1-R cells were highly resistant against cell death and protein synthesis inhibition by various anti-mesothelin RITs, including RG7787, a novel de-immunized RIT optimized for clinical use [19] but not against HB21(Fv)-PE, an immunotoxin targeting the transferrin receptor.

Previously reported mechanisms of resistance to the anti-CD22 *Moxetumomab pasudotox* RIT are directly linked to silencing or deletion of *DPH* genes, which prevents EF-2 ADP-ribosylation and subsequent protein synthesis inhibition [5–7]. This was not the case in KLM-1-R, where EF-2 ADP-ribosylation is normal. The higher EF-2 levels in resistant cells could imply that it takes the toxin longer to inhibit protein synthesis to the same extent as in KLM-1, but this is unlikely to cause the observed profound resistance, since even prolonged incubation with anti-mesothelin RITs gave virtually no cell death in the resistant cells, and KLM-1 and KLM-1-R were equally sensitive to HB21(Fv)-PE40, which also kills through EF-2 inactivation. The high sensitivity of KLM-1-R to HB21(Fv)-PE40 also indicates that the resistance is not associated with an aberration of the apoptotic machinery [20–22], and shows that the resistance is specific to anti-mesothelin RITs.

Mesothelin expression was partially down-regulated in KLM-1-R, resulting in a 4- to 5-fold lower cellular uptake of RG7787 compared to KLM-1. This provides one potential explanation for the observed resistance in KLM-1-R. Mesothelin overexpression has been associated with increased cell growth in PDAC cell lines [40]. However, despite the partial loss of mesothelin, KLM-1-R cells still grew significantly faster than KLM-1. We do not consider the lower mesothelin expression to be the cause of this increased proliferation, but rather hypothesize that it is linked to the 2-fold higher EF-2 levels in KLM-1-R. The increase in catabolic cell state was also reflected in the strong up-regulation of transcription and translation observed in the RNA sequencing analysis for KLM-1-R. The enhanced proliferation of KLM-1-R suggests cells changed and acquired resistance during RIT treatment. Since the resistant cells were not obtained via clonal selection, we cannot exclude the possibility that the KLM-1-R cell line is a polyclonal mixture of resistant cells, where each group of cells would display a different mechanism of resistance. However, none of the currently generated data support the presence of such mixture; flow cytometry analyses of resistant cells, e.g., display a single population with a uniform decrease in mesothelin surface expression and a homogeneous forward and side scatter profile. Further research is required to fully elucidate this possibility.

The *MSLN* gene is frequently hypomethylated in various patient tumors and cell lines, including PDAC, with the extent of methylation correlating with expression and shed mesothelin levels [35–37]. Hypomethylation of CpG sites typically increases gene transcription, whereas hypermethylation is associated with transcriptional silencing [34]. Aberrant methylation can contribute to the emergence of drug resistance in cancer cells [41]. Hypermetyhylation of *DPH1* and *DPH4* genes, e.g., is responsible for resistance against *Moxetumomab pasudotox* in leukemic cell lines [5, 7]. The deregulation of gene expression by CpG methylation can be reversed using DNA methyltransferase inhibitors such as AZA. In KLM-1-R, this compound significantly increased the surface expression of mesothelin. We analyzed an exploratory set of CpGs located in the promoter region of mesothelin and found that in KLM-1-R, these were subject to a significantly higher degree of methylation than in KLM-1. The hypermethylation in KLM-1-R was not absolute, which is expected considering that the resistant cells still expressed a moderate amount of mesothelin. Despite the limited number of analyzed CpGs, the overall results clearly link the partial down-regulation of mesothelin in KLM-1-R to hypermethylation. In addition, RNA sequencing expression profiles of KLM-1 and KLM-1-R matched with GSEA data sets indicated a more general state of hypermethylation in the resistant cells, supporting the impact of methylation RIT sensitivity.

There are several indications that the decrease in mesothelin is not the sole cause of resistance. AZA increased mesothelin expression in KLM-1-R to approximately 60% of the original levels in KLM-1, but cells were still about 180-fold more resistant to RG7787. In addition, several (PDAC) cell lines with a surface mesothelin similar to that of KLM-1-R or KLM-1-R-AZA have subnanomolar RG7787 IC_50_s [19], indicating that this amount of mesothelin expression is not necessarily a limiting factor. Furthermore, the introduction of *MSLN* in KLM-1-R restored the original sensitivity to RG7787, but was associated with a 5-fold overexpression of mesothelin compared to KLM-1, suggesting a significantly higher RG7787 uptake was needed to reach a similar sensitivity in KLM-1-R-*Msln*. These discrepancies hint that, in addition to the lower mesothelin, RIT trafficking might be a limiting factor in KLM-1-R. Indeed, we recently found that RIT cytotoxicity can depend on the toxin’s intracellular itinerary [42]. RNA sequencing data provided additional support for this hypothesis, with pathway analyses demonstrating differential expression in KLM-1-R of genes that are linked to intracellular transport. It is, however, currently unclear which transport-related genes could be considered the main drivers of such resistance mechanism. RIT trafficking has several uncertainties and is difficult to study, in part because of the few molecules actually trafficking to the cytosol. Given this complexity, this is beyond the scope of the current study and is subject to further investigations. Additional research is also required to establish whether the RIT resistance mechanisms herein described are common in mesothelin-expressing epithelioid cancer cell lines, and whether these aberrations can be found *in vivo*.

In conclusion, we isolated PDAC cells resistant to anti-mesothelin RITs. The resistance is linked to a methylation-associated decrease in mesothelin and subsequent low uptake of the RIT. Significant mesothelin overexpression and subsequent higher RG7787 uptake are required to reach the original cytotoxicity in resistant cells, hinting that RIT trafficking is also a limiting factor. Both the aberrations in methylation and intracellular transport were supported by gene expression analyses of the parental and resistant KLM-1 cells.

## Supporting Information

S1 Fig Microscopic evaluation of SS1-LR-GGS treatment in KLM-1. KLM-1 cells were treated for 72 hrs with 1 μg/ml SS1-LR-GGS. Bright-field pictures (10X) were taken at the day SS1-LR-GGS was added, 24 and 72 hrs later (after washing out the dead cells) from identical locations in the wells at each time point. A series of representative pictures is shown.
(PPTX)

S2 Fig Microscopic appearance, flow cytometry forward and sideward scatter, and *in vitro* growth of KLM-1 and KLM-1-R. **A**: Bright-field microscopic pictures (10X) of KLM-1 and resistant KLM-1 (KLM-1-R) show a similar appearance for both cell lines. **B:** KLM-1 and KLM-1-R cells match on the forward and sideward scatter profiles, indicating similar cell size and granularity. **C**: KLM-1-R cells grow significantly faster than KLM-1 starting from day 2 (*p* < 0.0001). 1 x 10^5^ cells were seeded and viable cells were counted in triplicate for the subsequent 6 days. Data is the average of two independent experiments.
(PPTX)

S3 Fig 147-bp nucleotide sequence in the mesothelin promoter region. The methylation status of the CGs (in bold) is analyzed by bisulfite pyrosequencing.
(PDF)

S4 Fig Gene set enrichment analysis database analysis reveals a hypermethylated state in KLM-1-R. RNA sequencing analysis on KLM-1 and KLM-1-R cells demonstrated significant changes in methylation patterns as shown by Qlucore’s functional analysis based on gene set enrichment analysis (GSEA) genes. The GSEA set “missiaglia_regulated_by_methylation_dn”, generated by treating PDAC cell lines with AZA [39], showed high similarity to our data. Of the 122 down-regulated genes in this GSEA, 97 (80%, in green) were also down-regulated in KLM-1-R, whereas 20 genes (16%, in red) were up-regulated and 5 genes (4%) were not overlapping.
(PPTX)

S1 Table Primer sequences for RT-qPCR. (DOCX)

S2 Table List of differentially up- or down-regulated genes in KLM-1-R versus KLM-1-R as determined by RNA sequencing analysis. (XLSX)
