# Supplementary figures and images for: Methylation-Associated Partial Down-Regulation of Mesothelin Causes Resistance to Anti-Mesothelin Immunotoxins in a Pancreatic Cancer Cell Line
Source: PLoS One. 2015 Mar 24;10(3):e0122462. doi: 10.1371/journal.pone.0122462 (PMC4372481; doi:10.1371/journal.pone.0122462)

## Slide 1
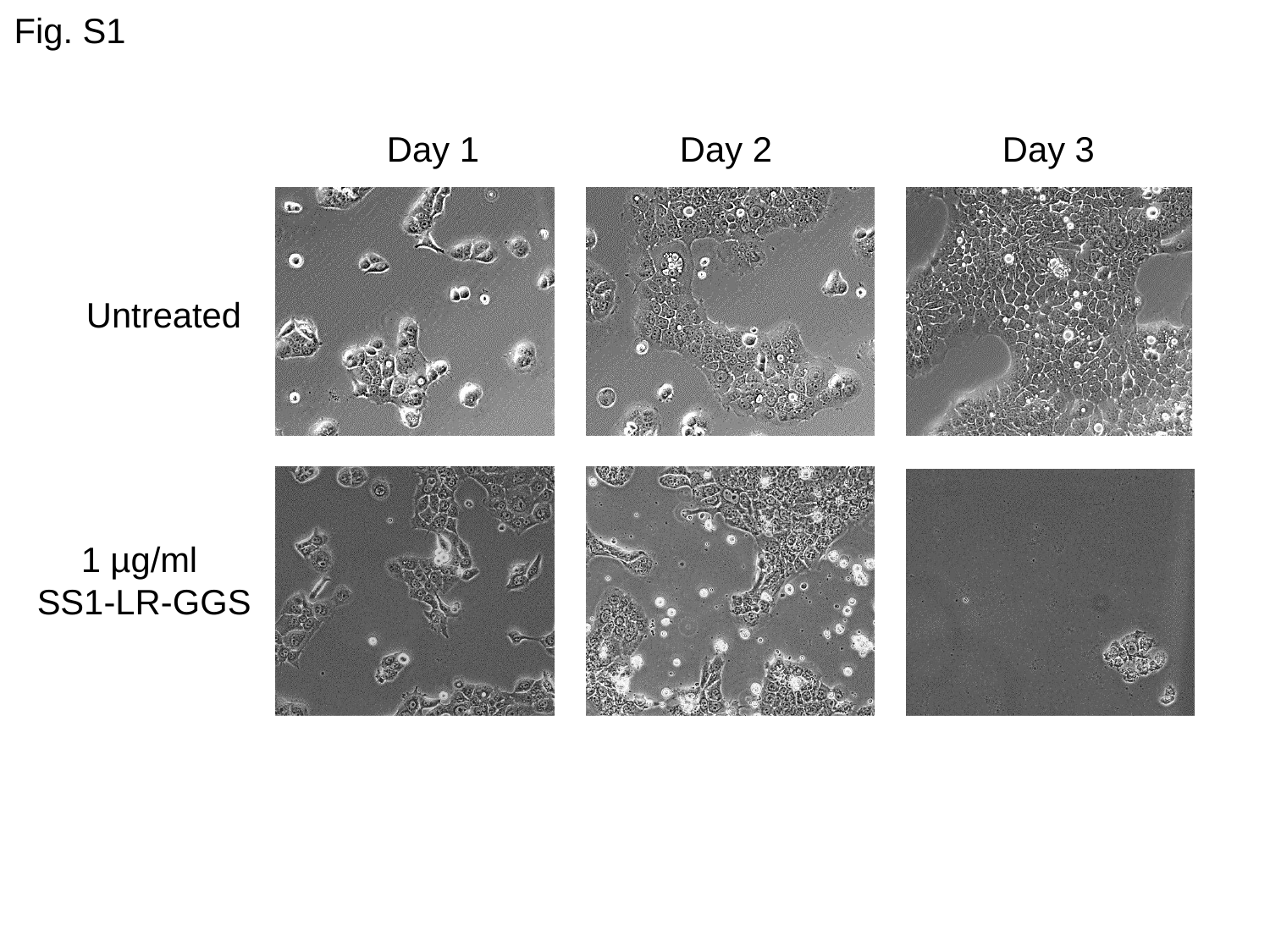

Fig. S1
 Day 1		 Day 2		Day 3
Untreated
1 µg/ml
SS1-LR-GGS

Supplement: S1 Fig — KLM-1 cells were treated for 72 hrs with 1 μg/ml SS1-LR-GGS. Bright-field pictures (10X) were taken at the day SS1-LR-GGS was added, 24 and 72 hrs later (after washing out the dead cells) from identical locations in the wells at each time point. A series of representative pictures is shown. (PPTX) [file pone.0122462.s001.pptx]

## Slide 1
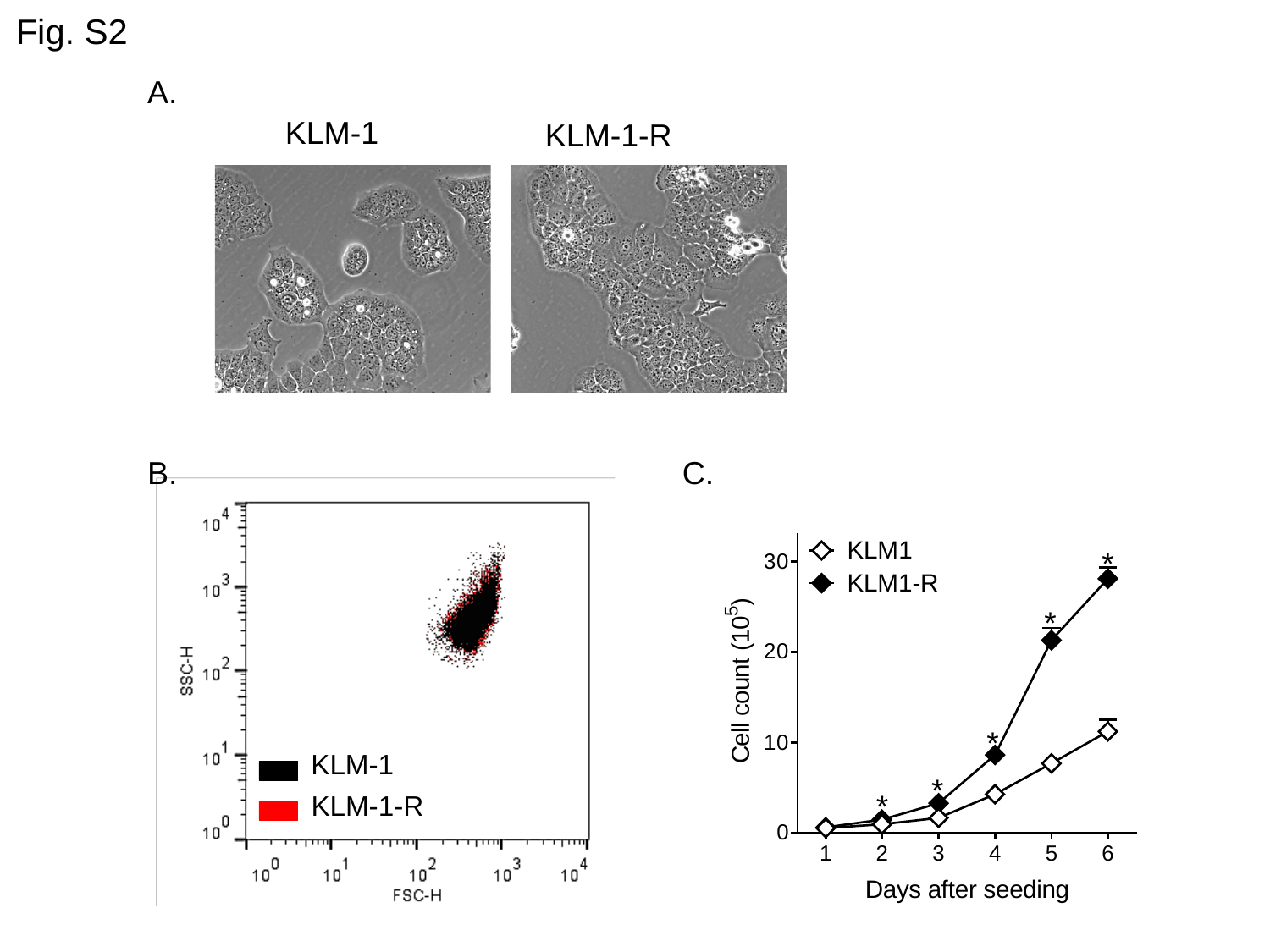

Fig. S2
A.
B. C.
KLM-1
KLM-1-R
KLM-1
KLM-1-R

Supplement: S2 Fig — A: Bright-field microscopic pictures (10X) of KLM-1 and resistant KLM-1 (KLM-1-R) show a similar appearance for both cell lines. B: KLM-1 and KLM-1-R cells match on the forward and sideward scatter profiles, indicating similar cell size and granularity. C: KLM-1-R cells grow significantly faster than KLM-1 starting from day 2 (p < 0.0001). 1 x 10^5^ cells were seeded and viable cells were counted in triplicate for the subsequent 6 days. Data is the average of two independent experiments. (PPTX) [file pone.0122462.s002.pptx]

## Slide 1
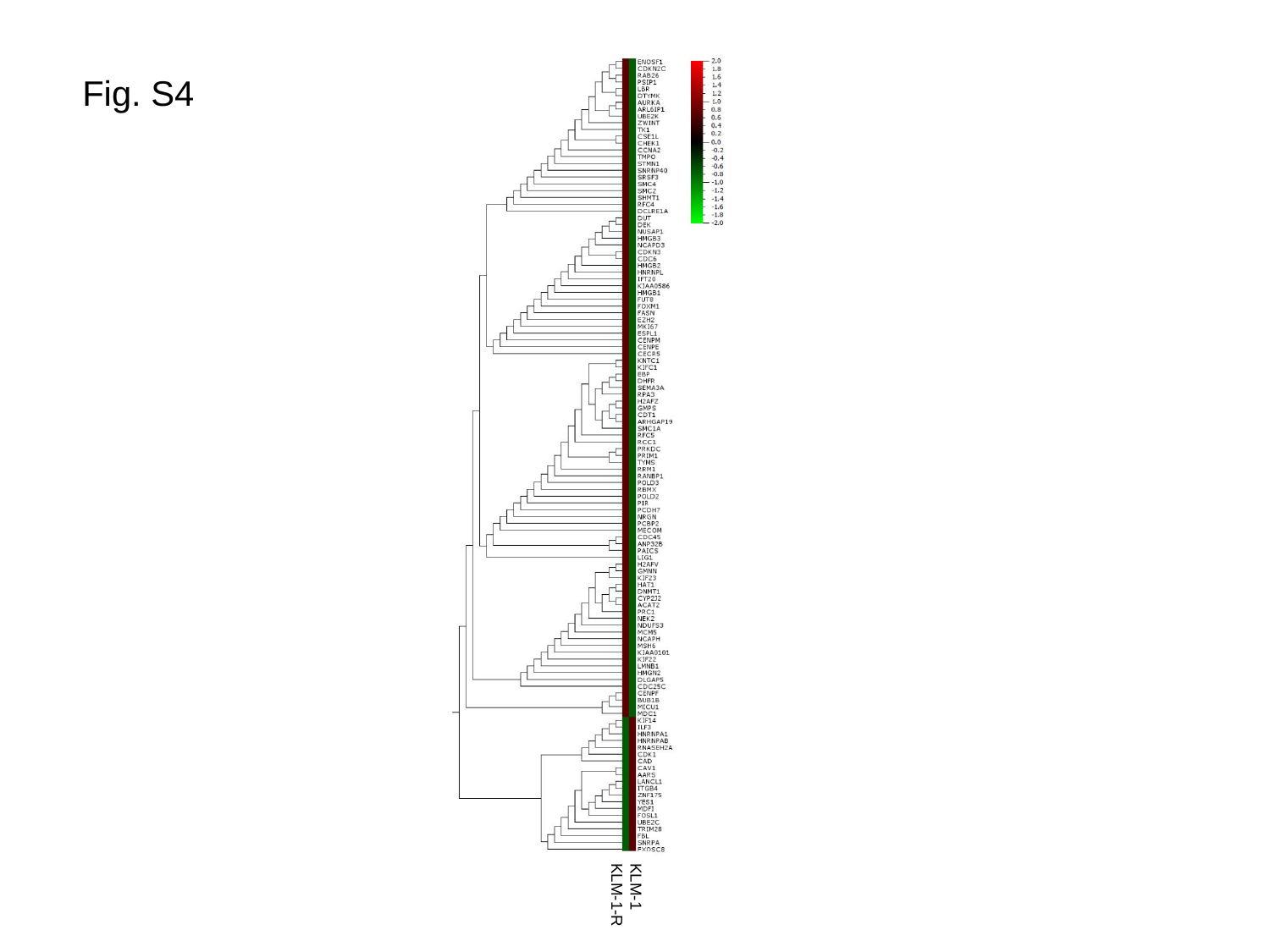

KLM-1
KLM-1-R
Fig. S4

Supplement: S4 Fig — RNA sequencing analysis on KLM-1 and KLM-1-R cells demonstrated significant changes in methylation patterns as shown by Qlucore’s functional analysis based on gene set enrichment analysis (GSEA) genes. The GSEA set “missiaglia_regulated_by_methylation_dn”, generated by treating PDAC cell lines with AZA [39], showed high similarity to our data. Of the 122 down-regulated genes in this GSEA, 97 (80%, in green) were also down-regulated in KLM-1-R, whereas 20 genes (16%, in red) were up-regulated and 5 genes (4%) were not overlapping. (PPTX) [file pone.0122462.s004.pptx]
