# Supplementary material for: Methylation-Associated Partial Down-Regulation of Mesothelin Causes Resistance to Anti-Mesothelin Immunotoxins in a Pancreatic Cancer Cell Line
Source: PLoS One. 2015 Mar 24;10(3):e0122462. doi: 10.1371/journal.pone.0122462 (PMC4372481; doi:10.1371/journal.pone.0122462)
Supplement: S3 Fig — The methylation status of the CGs (in bold) is analyzed by bisulfite pyrosequencing. (PDF) [file pone.0122462.s003.pdf]

Fig. S3

TGTATTTTTAGTAGAGA**CG**GGGTTTCAC**CG**TGTTAGCCAAGATGGTCT**CG**ATCT
CCTGACCTCATGATCTGCCTGCCT**CG**GCCTCCAAAAGTGCTGGGATTACAGG**C**
GTGAGTCACTG**CG**CC**CG**GCATTTTTTTTTTTTTTTTTTTT
