# Supplementary material for: Methylation-Associated Partial Down-Regulation of Mesothelin Causes Resistance to Anti-Mesothelin Immunotoxins in a Pancreatic Cancer Cell Line
Source: PLoS One. 2015 Mar 24;10(3):e0122462. doi: 10.1371/journal.pone.0122462 (PMC4372481; doi:10.1371/journal.pone.0122462)
Supplement: S1 Table — (DOCX) [file pone.0122462.s005.docx]

**Table S1. Primer sequences for RT-PCR**

| **Gene** | **Sequences** |
| --- | --- |
| DPH1 | F: 5’-GAAGGTGGCCTTGCAAATGCC  R: 5’-TGGCCGTAGTGCACCAAGAAG |
| DPH2 | F: 5’- GCAGCCTCATTCCTTAGTTCC  R: 5’-CCGCTTCCCTCATCCTCATAG |
| DPH3 | F: 5’-AGAAATCTCATTGCCATTTGGG  R: 5’-CATCAAGGGTGTCTGTTCGTGT |
| DPH4 | F: 5’-CCAAGGATGAAGCGGAAGAAG  R: 5’-GCAAATAATCGGGCTGACAAATAA |
| DPH5 | F: 5’-CCAAGCAGCCCAGCAGCTTCT  R: 5’-CAAGTCCACAGTGCACATTTGC |
| DPH7 | F: 5’-CCCGACTCGCTGGTGTATG  R: 5’-GGTGTTGGCAACTCGCTTG |
| Mesothelin | F: 5’-tcctgttcctgctcttcagcc  R: 5’-acacgggaagccaaggagttg |
| ß-actin | F: 5’-GCCAGCCAGGTCCAGAC  R: 5’-AGGCCAACCGCGAGAAGAT |
